# Effect of Crystallinity of Polyethylene with Different Densities on Breakdown Strength and Conductance Property

**DOI:** 10.3390/ma12111746

**Published:** 2019-05-29

**Authors:** Dawei Li, Liwei Zhou, Xuan Wang, Lijuan He, Xiong Yang

**Affiliations:** 1 College of Electrical and Electronic Engineering, Harbin University of Science and Technology, Harbin 150080, China; deardawei4li@163.com (D.L.); 15776629484@163.com (L.Z.); YangXiong@163.com (X.Y.); 2 Key Laboratory of Engineering Dielectrics and Its Application Harbin University of Science and Technology, Harbin 150080, China; 3 Rongcheng College of Harbin University of Science and Technology, Rongcheng 264300, China

**Keywords:** polyethylene, crystallinity, breakdown strength, conduction current, SCLC

## Abstract

In order to study the effects of the crystallinity of polyethylene with different densities on breakdown strength and conductance properties, this paper mainly tests the X-ray diffraction (XRD), different scanning calorimeter (DSC), direct current (DC) breakdown and conductance properties of low-density polyethylene (LDPE), linear low density polyethylene (LLDPE), medium density polyethylene (MDPE), and high-density polyethylene (HDPE), and further analyzes the experimental results separately. The results show that an increase in the density of polyethylene leads to the continuous improvement of crystallinity, and an increase in crystallinity causes a significant decrease in the conduction current at the same field strength. The field strength corresponding to the two turning points in the conductance characteristic curve increases simultaneously.

## 1. Introduction

Polyethylene is a partially crystalline solid whose properties are highly dependent on the relative content of the crystalline phase and amorphous phase, i.e., crystallinity. Polyethylene is a polymer polymerized from monomeric ethylene. It is widely used in the insulating material of power cables due to its symmetrical molecular structure and no polar groups, which makes it has excellent electrical and mechanical properties [1,2]. Classified according to different polymerization methods, polyethylene can be classified into linear low-density polyethylene (LLDPE), low-density polyethylene (LDPE), medium density polyethylene (MDPE), and high-density polyethylene (HDPE). Linear low-density polyethylene has a regular short-chain structure, although its crystallinity and density are similar to those of low-density polyethylene, the intermolecular force is larger. The macromolecules of low-density polyethylene have many branches and cannot be closely and regularly arranged with each other, and its branching degree is high. The medium–high density polyethylene are linear macromolecules with a low branching degree and regular structure [3].

As an insulating material, polyethylene is easy to cause electrical trees under the effect of high electrostatic voltage field, which eventually leads to insulation breakdown [4]. At present, domestic and foreign scholars generally agree that the space charge effect plays an important role in the insulation aging process [5,6]. The existing research has mainly used space charge limited current (SCLC), Schottky, Poole-Frenkel and hopping conductivity to explain the conductance mechanism of pure polyethylene or polyethylene nanocomposites in high field strength regions (non-ohmic regions). Some scholars believe that the conductivity mechanism of polyethylene under high field strength is not dominated by a single conductance mechanism [7,8], but a variety of conductance mechanisms [9]. Some scholars believe that the charge transport mechanism of doped nano-polyethylene in high field strength region (non-ohmic region) is dominated by ion hopping conductance, and it can be deduced from the formula that the ion jump distance increases with increasing temperature [10,11]. Further, they pointed out the “pre-electric stress” effect of polymer nanocomposites under a high electric field [12,13]; others believe that it is dominated by electronic hopping conductance, and the jump distance decreases with increasing temperature [14].

Many scholars have done a lot of research on the conductance property of polymer nanocomposites, but most of them have studied the modification of low density polyethylene matrix nano [15,16,17]. There are very few studies on the conductance property of polyethylene with different densities. In addition, due to the complexity of the structure of polymer materials, there is currently a lack of sufficient understanding of the conductance mechanism of polyethylene. Based on the existing research on polyethylene and polyethylene nano-polyethylene [18,19], this paper studied the effects of different crystallinity on the DC breakdown strength and conductance property of different density polyethylene. The effects of crystallinity of polyethylene with different densities on its conductance property were discussed by XRD and DSC.

## 2. Experiment

### 2.1. Experimental Materials

The paper selected linear low-density polyethylene (LLDPE), low-density polyethylene (LDPE), medium density polyethylene (MDPE) and high-density polyethylene (HDPE) as test materials. The LLDPE model is LLDPE7042 (Jilin petrochemical compan, Jilin, China) with a density of 0.922 g/cm^3^. The model of LDPE is LD200BW (Beijing Yanshan branch of Sinopec, Beijing, China) with a density of 0.918 g/cm^3^, and the model of MDPE is MDPE157 (Beijing Yanshan branch of Sinopec) with a density of 0.935 g/cm^3^. The HDPE model is DMDA 8008 (Daqing Petrochemical Company, Daqing, China) with a density of 0.954 g/cm^3.^

### 2.2. Sample Preparation

The LDPE sample was pressed by a plate vulcanization machine (Harper Electric Technology Co., Ltd, Harbin, China) at 110 °C, preheated for 5 min before pressurization, then pressurized at 5 MPa every 5 min, and finally pressed at a maximum pressure on 15 MPa. The thickness of the sample used to test the DC breakdown was 60 μm, and the thickness of the sample used to test the conductance was 50 μm. The samples of LLDPE, MDPE, and HDPE were prepared in the same manner, but the pressing temperature was changed into 150 °C. All the samples were made by depositing aluminium as electrodes on the vacuum membrane plate machine. Finally, the prepared electrode samples were placed in a vacuum drying oven for 24 h, and the short-circuit temperature was 50 °C.

### 2.3. Different Scanning Calorimeter DSC Test

The thermal properties of polyethylene with four different densities were tested by differential scanning calorimeter. During the experiment, 0.005–0.007 g samples were weighed and placed in aluminum crucibles, protected by high-purity nitrogen, and the heating and cooling temperature rate were set to 10 °C/min. The sample was first heated to 200 °C to completely melt, eliminating the influence of thermal history, and then cooled to 40 °C to obtain a crystallization process curve, and then heated to 200 °C to obtain a melting curve.

### 2.4. XRD Test

Phase analysis of polyethylene with four different densities was carried out using an X-ray diffractometer (X’pert, Leeman Company, Rome, Italy). The X-ray source was Cu Kα, the tube voltage was set to 40 kV, the tube flow was set to 40 mA, the phase analysis was performed in the *θ*–2*θ* scanning mode, the step size was set to 0.05°, and the time constant was 1 s. The *θ*–2*θ* scanning mode is used for fine scanning, the step size is set to 0.02°, and the time constant is 20 s.

### 2.5. Conductivity Test

**Figure 1:**
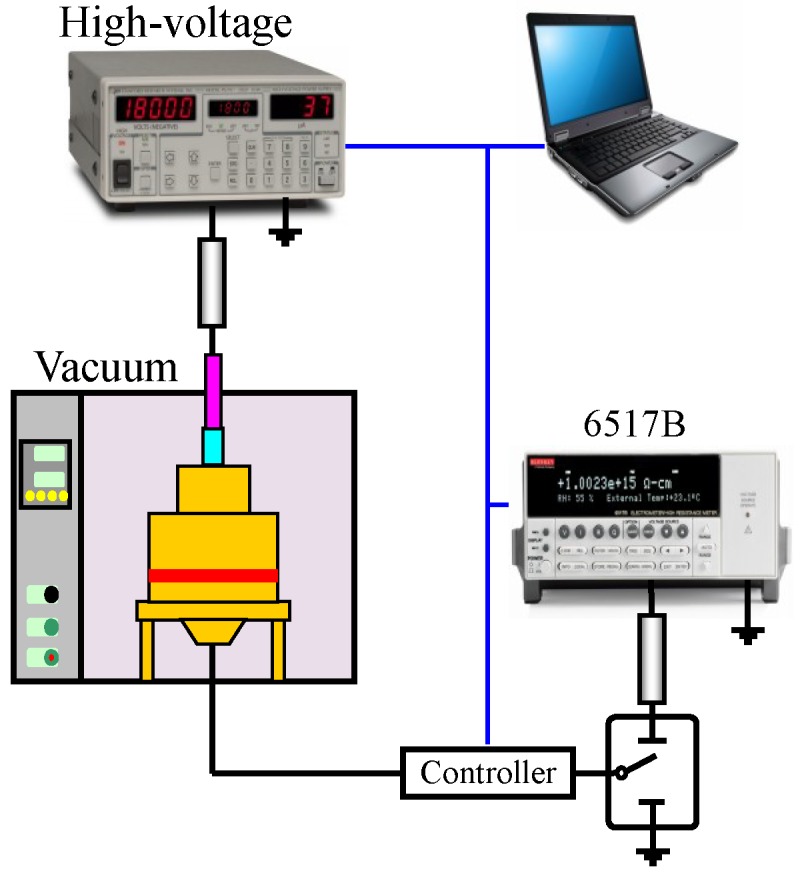
Conductivity test schematic.

The schematic diagram of the test system for conduction current is shown in Figure 1. Using the high resistance meter (minimum theoretical measurement accuracy is 10^−15^ A) and homemade three-electrode system (measuring electrode diameter is 25 mm), the conductance properties of pure LLPE, LDPE, MDPE and HDPE with a thickness of 50 μm were measured at the field intensity of 5–200 kV/mm for 30 min in a vacuum environment (pressure in the vacuum chamber is 0.1 MPa). Using computer to realize high-voltage DC power (Stanford Research Systems, Inc., Stanford, CA, USA), 6517B high resistance meter and controller’s boost automatic control, automatic data acquisition and storage, and automatic control of protection circuit. Each pressor step of the voltage source is 250 V, and the pressurization time is 30 min. When the time of pressurization reaches 30 min, the controller controls the high voltage relay to operate, and the 6517B high resistance meter is connected to the measuring circuit for a current test of 30 s. Collect 10 data per second, and finally select the average value as the value of conductance current at this voltage. After the measurement is finished, the controller adjusts the high-pressure vacuum relay to operate, and the 6517B high-resistance meter is continuously disconnected, and then re-pressurized, thereby repeating.

## 3. Experiment Results and Discussion

### 3.1. Polyethylene Melting Characteristics

**Figure 2:**
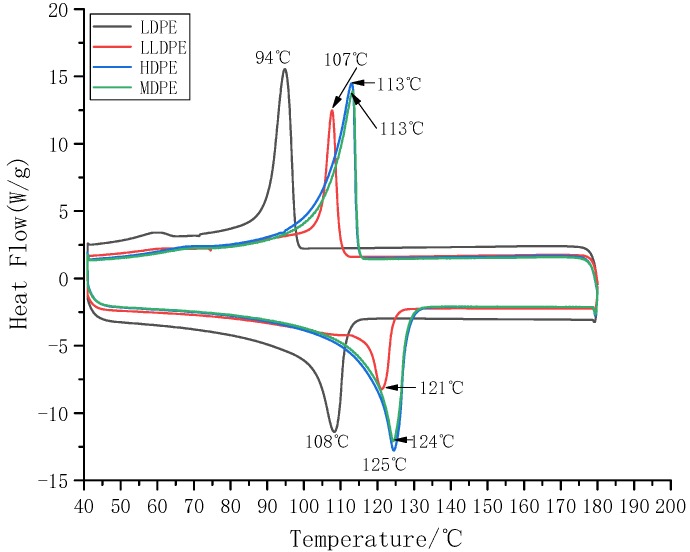
DSC curves of four kinds of polyethylene. (Low-density polyethylene, LLDPE; low-density polyethylene, LDPE; medium density polyethylene, MDPE; high-density polyethylene, HDPE).

Figure 2 shows the melting characteristics of four kinds of polyethylene. It can be seen from Figure 2 that the melting temperature of LDPE is the lowest and the melting peak is small; the melting temperature of LLDPE is higher than that of LDPE, the melting peak is lower than that of LDPE, the melting temperature of MDPE is higher than that of LLDPE, and the melting peak is higher than that of LLDPE; The highest melting temperature and melting peak of HDPE are very similar to MDPE, and the melting peak area is slightly larger than MDPE. Crystallinity (*X_c_*) can be used to characterize the ratio of the crystalline part of the semi-crystalline polymer, and the calculation of *X_c_* is shown as:

(1) $$X_{c}=\frac{\Delta H_{m}}{\Delta H_{100}}\times 100\%$$

where, *ΔH*_m_ is the enthalpy absorbed by the test sample during the heating process, *ΔH*_100_ is the enthalpy absorbed by the sample during the crystallization-melting process.

The *ΔH*_100_ of polyethylene is 293 J/g [20,21]. The crystallinity of the four kinds of polyethylene was calculated by DSC test software and the results are shown in Table 1. It can be seen from Table 1 that the crystallinity of the four kinds of polyethylene is different, the crystallinity of LDPE is the lowest, the crystallinity of HDPE is the highest, and the crystallinity of MDPE is higher than that of LLDPE. And the crystallinity of LLDPE is slightly higher than LDPE, but the two are very similar. It can be seen that LLDPE and LDPE not only have a similar density but also have a similar crystallinity. At the same time, it is known from experiments that crystallinity increases with density.

### 3.2. Analysis of the Crystal Structure of Different Polyethylene

**Figure 3:**
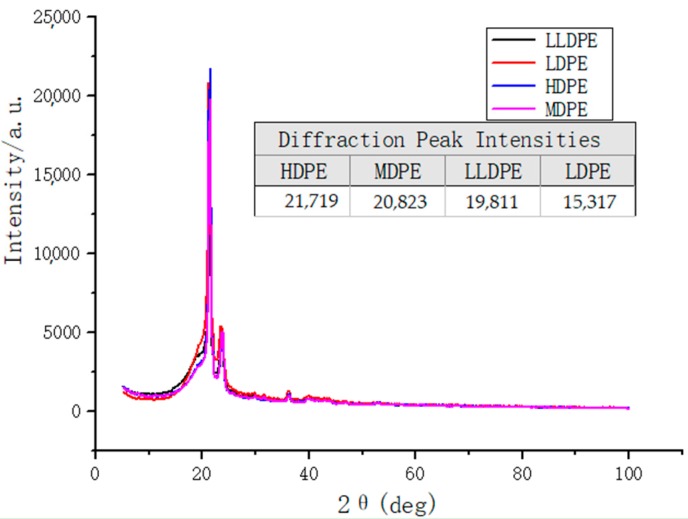
XRD images of four kinds of polyethylene.

Figure 3 shows that the four kinds of polyethylene with different densities has obvious diffraction peaks at nearly the same position 2*θ* = 21.42°, indicating that these four kinds of polyethylene have typical crystal structures. Moreover, from the image and data, it can be concluded that the diffraction peak intensities of the four kinds of polyethylene are arranged from small to large: HDPE, MDPE, LLDPE, LDPE. The crystallinity of the polymer is directly proportional to the diffraction peak intensity of the XRD, so it can be concluded that the crystallinity of the four polymers is in order from large to small: HDPE, MDPE, LLDPE, LDPE. The image results of XRD are completely consistent with the results obtained through the DSC data calculation, so the crystallinity of four kinds of polyethylene with different densities can be determined in this experiment.

### 3.3. DC Breakdown Strength Test

**Figure 4:**
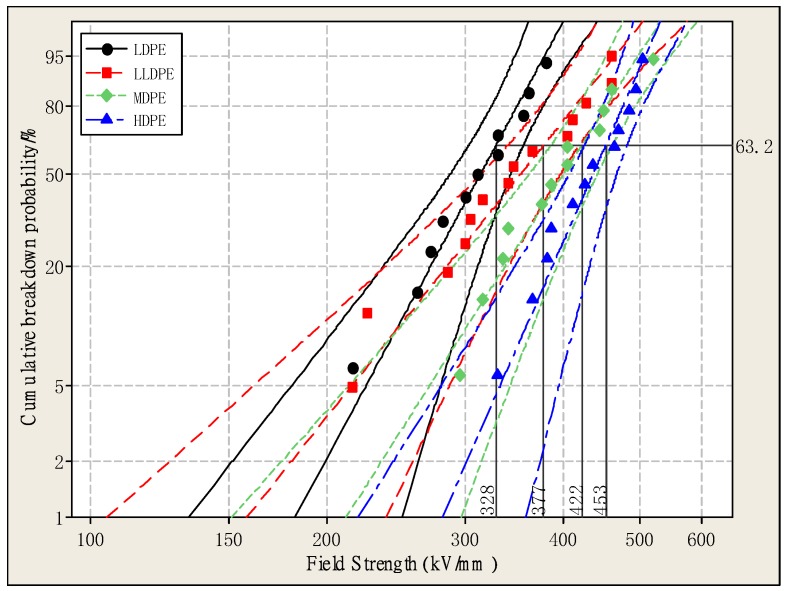
The Weibull plots of the direct current breakdown strength of four kinds of polyethylene.

Figure 4 is a Weibull distribution of DC breakdown strength for four kinds of polyethylene with different densities. The shape parameters and characteristic breakdown strength of four kinds of polyethylene are shown in Table 2. The shape parameter indicates the dispersion of the breakdown data, the characteristic breakdown strength represents the electric field strength when the overall sample reaches a 63.2% breakdown probability. It can be seen from Table 2 that the breakdown strength of the four kinds of polyethylene is different, and the breakdown strength of LDPE is the lowest. The breakdown field strength of HDPE, MDPE, and LLDPE is 37.96%, 28.56% and 15.01% higher than that of LDPE, respectively. It can be concluded that the breakdown strength of the four kinds of polyethylene increases with the increase of crystallinity, mainly because the free volume of polyethylene becomes smaller as the crystallinity increases. Thereby, the free path of electrons is reduced, it becomes difficult for them to accumulate energy in the electric field, and the probability of electrons accelerating under the electric field is lowered so that the breakdown strength is correspondingly increased [22]. It can also be seen from Table 2 and Figure 4 that the larger the shape parameter, the smaller the dispersion of the breakdown strength data, and the polyethylene exhibits more stable dielectric properties.

### 3.4. DC Conductance Characteristics Analysis

#### 3.4.1. Conduction Current Theory

##### (a) Traps and Space Charge

There are many localized states in the forbidden band gap of the polymer. These localized states can capture the carriers in the material to form a space charge, which acts as a trap, so the localized state is also called a trap. Space charge is sometimes referred to as trapped charge. Traps are mainly caused by crystal imperfection, which is caused by structural defects or impurities, or both. Traps are roughly divided into two types, mainly formed by structural defects or chemical defects. It is generally believed that discrete trapping levels are associated with chemical impurities doped in the lattice, and quasi-continuous trapping levels are related to imperfections of crystal structure [23].

##### (b) Space Charge Limited Current (SCLC)

**Figure 5:**
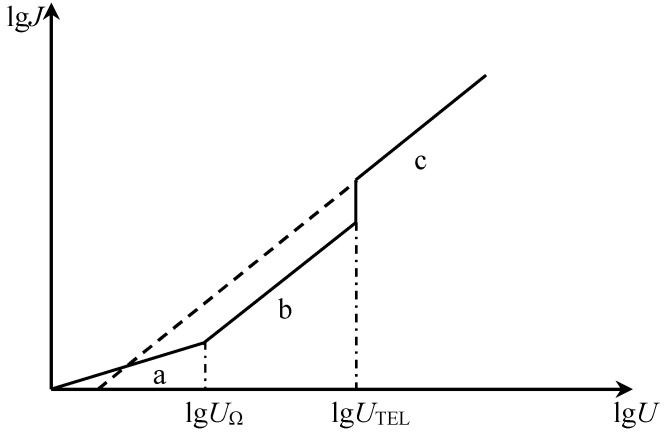
Relationship between space charge limited current in dielectrics and the applied voltage. (a region—Linear region is Ohmic conduction current region; b region—Calder’s law region of space charge limited current when trapped; c region—Calder’s Law region with trap filling or without traps).

The current-voltage characteristics of the dielectric comply with Ohm’s law at low electric fields, i.e., at the beginning of pressurization. When the voltage (or electric field intensity) reaches a certain value *U_Ω_* (or *E_Ω_*), the concentration of the injected carriers increases, accumulating a large amount of space charge, causing the space charge limited current. So that the current flowing through the dielectric is transformed from the region of ohmic current to the region of space charge limited current. When the field strength applied to the material exceeds the breakover field strength, a large accumulation of carriers and space charge limited current will occur in the insulation, which may cause various aging conditions. Thus, the breakover voltage *U_Ω_*(or electric field intensity *E_Ω_*) is sometimes referred to as the electric degradation threshold of the dielectric material, which is the ideal situation without traps. There are inevitably various traps for the actual dielectric materials as mentioned above. When there are traps, the trapping of the injected charge makes the breakover voltage much larger than when there is no trap, and the current is made smaller. As the voltage U applied to the material increases, the amount of injected charge increases and the traps in the material are gradually filled. The traps are filled when the voltage reaches a certain value U_m_, and the injected electrons will no longer cause trapping so that the current in the insulation increases so sharply that it turns to the region of space charge limited current without traps. At this time, the density *J* of the space charge limited current follows Calder’s law with trap filling or without traps, as shown in Figure 5 [24].

The expression of the density of the space charge limited current is shown as:

(2) $$J=(\frac{9}{8}\varepsilon_{r}\varepsilon_{0}\mu \frac{U^{2}}{d^{3}})\theta$$

where, ε_0_ is the vacuum dielectric constant, ε_r_ is the relative dielectric constant, *μ* is the permeability, *d* is the dielectric thickness, *θ* is the control parameter of the trap. *θ* = *n*/(*n* + *n_t_*)*,* which is the ratio of the free carrier concentration to the total carrier concentration. *n_t_*is the trapped carrier concentration, *n* is the free carrier concentration, since *n_t_*>> *n*, *θ* ≈ *n*/*n_t_*, usually *θ* *≤* 10^−7^ [8,20].

The breakover voltage *U_Ω_*of the space charge limited current can be expressed as Equation (3), *n_t_*is shown as:

(3) $$U_{\Omega }=\frac{8end^{2}}{9\varepsilon_{r}\varepsilon_{0}\theta }$$

(4) $$n_{t}=\frac{9\varepsilon_{r}\varepsilon_{0}U_{\Omega }}{8ed^{3}}$$

where, *e* is the amount of electron charge.

Equation (5) can be obtained after taking a logarithm of both sides of Equation (2):

(5) $$\ln J=\ln\frac{9\varepsilon_{r}\varepsilon_{0}\mu }{8d^{3}}+2\ln U$$

It can be seen from Equation (5) that the current density and the applied voltage of the SCLC is linear in the double logarithmic coordinate with a slope of 2.0.

#### 3.4.2. Test Results and Analysis of Conduction Current

**Figure 6:**
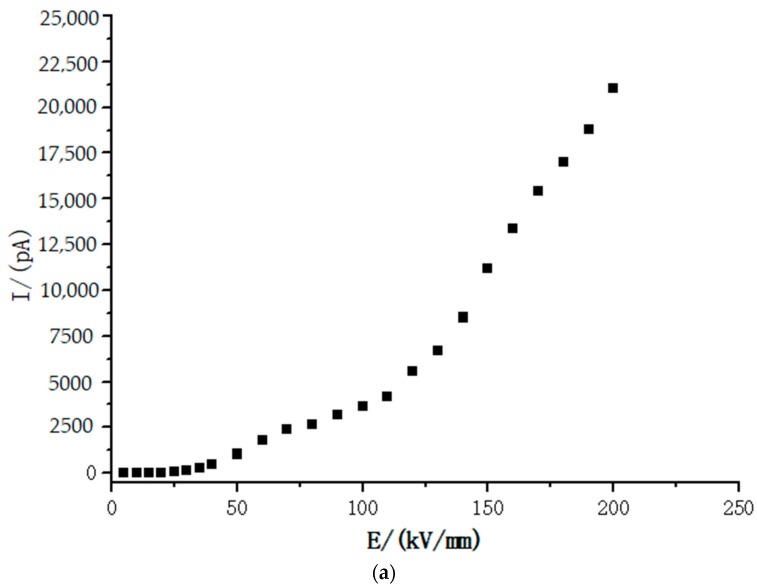

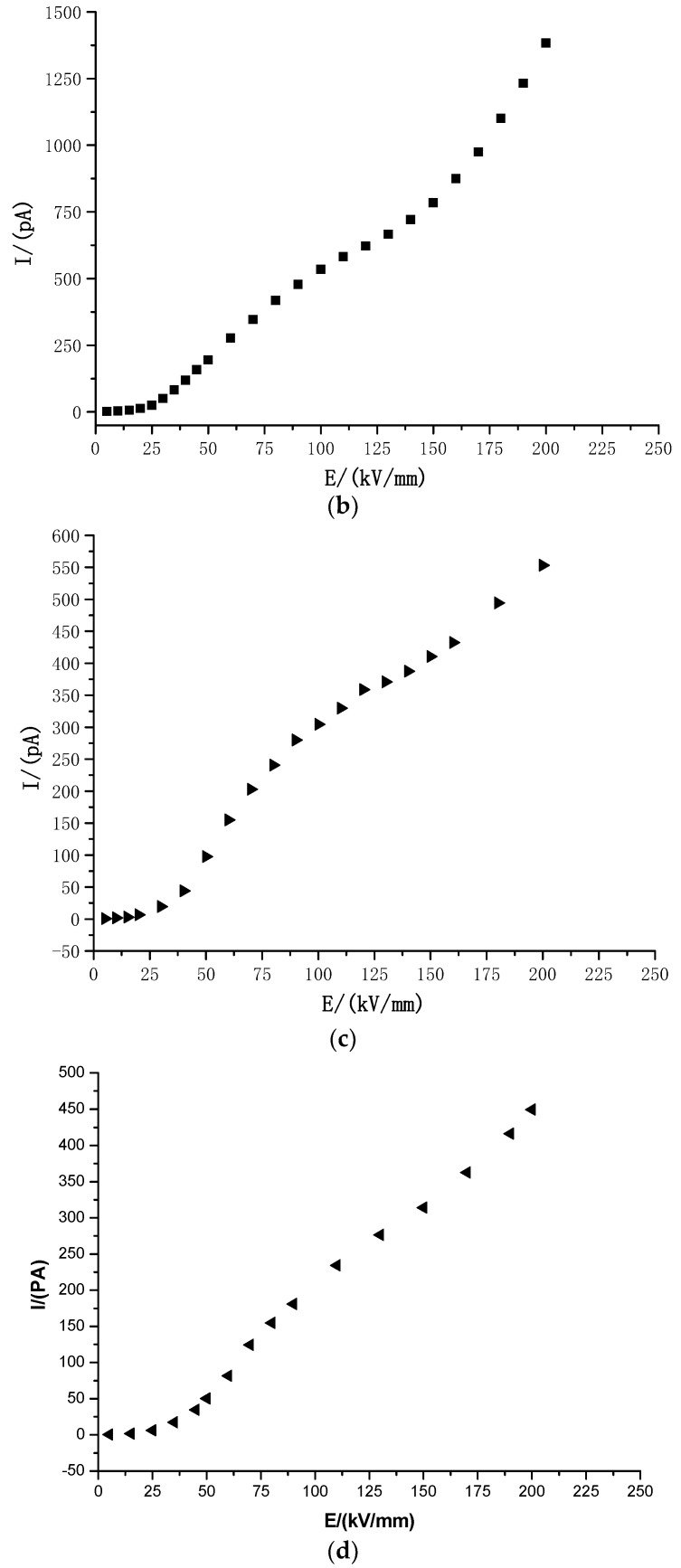
Conduction current characteristic curve of polyethylene with different densities. (**a**) LDPE; (**b**) LLDPE; (**c**) MDPE; (**d**) HDPE.

**Figure 7:**
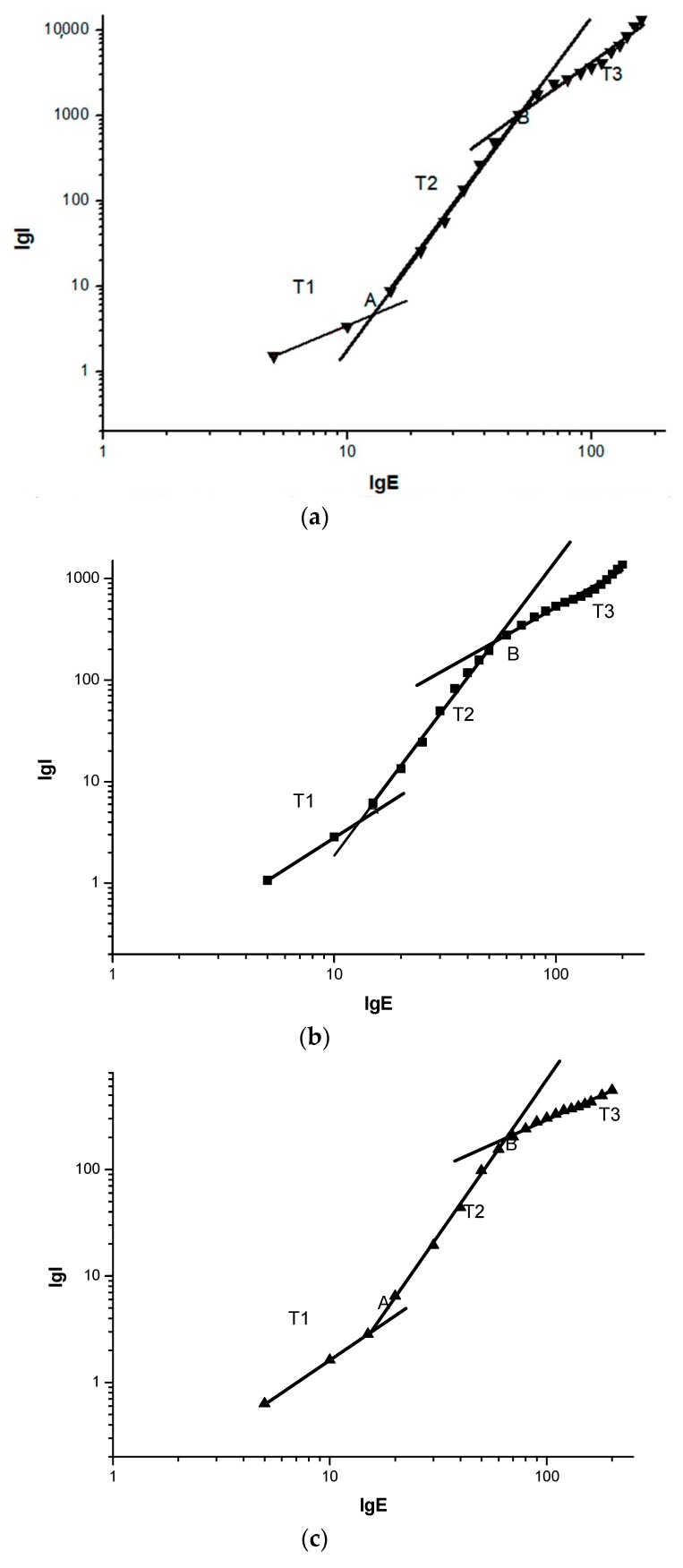

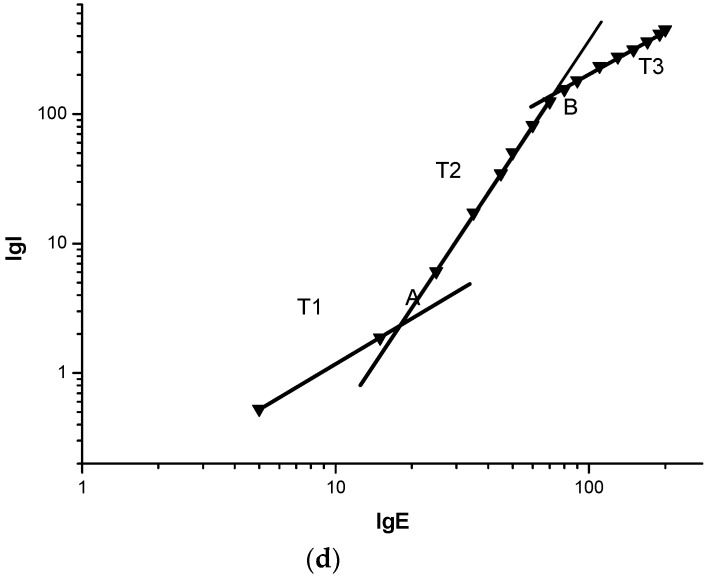
Logarithmic form fitting diagram of electrical conductivity flow of four different density polyethylenes. The logarithmic form of the conductance characteristic curve of (**a**) LDPE, (**b**) LLDPE, (**c**) MDPE, (**d**) HDPE.

Figure 6 shows the characteristics of the conduction currents of LDPE, LLDPE, MDPE, and HDPE at different densities. For the convenience of analysis, the experimental results are expressed in logarithmic form and piecewise fitting method is adopted. It can be seen from the fitting results in Figure 7 that the characteristic curves of the four kinds of polyethylene have two turning points, point A and point B, and three areas, namely T1 area, T2 area and T3 area. The slopes of the fitted straight lines for the T1, T2, and T3 regions are given in Table 3. The field strength corresponding to points A and B of the four kinds of polyethylene with different densities has been given in Table 4.

In addition, the values of conduction current corresponding to LDPE, LLDPE, MDPE, and HDPE are sequentially decreased in the same field strength in Figure 6, so that their conductivity is also sequentially decreased at the same field strength. The carrier mobility is related to the migration barrier and the skip distance. As the density of different polyethylene increases, the lamellae thickness, and convergence of polyethylene increase [25]. The compact degree of the molecular chains of MDPE, HDPE, LLDPE, and LDPE is weakened in turn, and the compactness of the lamellae is weakened in turn, so the height of the migration barrier is sequentially reduced. The skip distance is related to the lamellae thickness. As the crystallinity increases, the lamellae thickness increases and the average skip distance increases, so the carrier mobility decreases significantly as the density of polyethylene increases.

In Table 3, the slope of the T1 region, i.e., the low field strength region (ohmic region) is close to 1, which remains substantially unchanged. The curve slope in the T2 region, i.e., the high field strength region (non-ohmic region), decreases with increasing density, but the slopes are all greater than 2 on average. By studying the conductance mechanism of different kinds of polyethylene, Montanari believes that the curve slope in the logarithmic form of the high field strength region is greater than 2, and there is an effect of space charge limited current (SCLC) [26].

According to Table 4, the field strength at the first turning point A of the conductivity curves of the four kinds of polyethylene with different densities was: 12.76 kV/mm, 13.33 kV/mm, 15.68 kV/mm, and 18.37 kV/mm, respectively. It can be seen that as the density of polyethylene gradually increases, the electric field threshold from the ohmic zone to the non-ohmic zone also gradually increases. Some scholars believe that there are many local states in the band gap energy of polymers. These local states can trap carriers in materials to form space charges [27]. According to the theory of space charge limited current, the breakover voltage from the ohmic region to the space charge limited current region corresponds to the electric field intensity formed by a large accumulation of space charges. The reason for the data in Table 4 may be that LDPE has the largest conduction current, i.e., the most effective carriers. LLDPE, MDPE, and HDPE are sequentially reduced at the same field strength in the ohmic region. Therefore, LDPE may first start the accumulation of space charges. The field strengths at the turning point, which is the point B in the high field strength region (non-ohmic region), are 50.88 kV/mm, 53.61 kV/mm, 65.34 kV/mm, and 71.89 kV/mm, respectively. It can be seen that as the density of polyethylene increases, the field strength corresponding to point B continues to increase. Some scholars believe that the occurrence of turning points in high field strength regions (non-ohmic regions) may be due to tunneling effects in charge filling regions or high field strengths [28]. According to the theoretical analysis of the conductance characteristics of Figure 5, the slope of the characteristic curve of the trap filling region (C region) should be the same as the slope of the characteristic curve of the space charge limited current region. However, it can be seen from Table 3 that the slope of the characteristic curve of the T3 region is significantly smaller than that of the T2 region (SCLC region), which may be due to the fact that deeper traps in the dielectric material may be excited when the field strength is particularly high. Therefore, when the field strength reaches the second turning point, the carrier may be trapped by the deeper trap, which further causes the curve slope of the conductance characteristic to be much smaller in the T3 region than in the T2 region. In practical applications of engineering dielectrics, the T3 region in the conductance characteristic curve (non-ohmic region) at different field strengths is not a trap filling region. To prove whether the theoretical trap filling region exists, it is necessary to further explore and think experimentally.

## 4. Conclusions

In this paper, the XRD, DSC, DC breakdown and conductance characteristics at 5–200 kV/mm of four kinds of polyethylene with different densities (LDPE, LLDPE, MDPE, HDPE) were measured based on room temperature, and the main conclusions were as follows:

(1) For four kinds of polyethylene with different densities, their crystallinity increases with increasing density.
(2) With the increase of the crystallinity of four kinds of polyethylene with different densities, their corresponding breakdown strength also increased significantly. At the same time, the larger the shape parameter, the smaller the data dispersivity of the breakdown strength, and the polyethylene has more stable dielectric properties.
(3) In the test of conductivity characteristics, the electric threshold of LDPE, LLDPE, MDPE, and HDPE transitioning from the ohmic region (T1 region) to the non-ohmic region is gradually decreased, and the field strength at the turning point B of the non-ohmic region is also gradually decreased. This is because the conductivity of the four materials is gradually reduced, and the number of carriers at the same field strength is gradually reduced, so the largest number of carriers may reach the turning point first. It can be seen from the logarithmic form of the image I-E after piecewise fitting, the slopes of the conductivity curves of the four materials in the T1 region are close to 1, almost the same as the theoretical value. And the slope of the T2 region is greater than 2, indicating the existence of the SCLC effect in this region. The curve slope of the conductance characteristic in the T3 region is much lower than the curve slope in the T2 region. This may be because this region is not a trap filling region, and the further increase of the electric field intensity in the non-ohmic region excites the deeper trap of the dielectric material. The carriers fall into the trap again, resulting in the curve slope of the conductance characteristic of the T3 region is smaller than that of the T2 region.

## Figures and Tables

**Table 1:** Crystallization and melting process parameters of four kinds of polyethylene.

Sample	T_m_/(°C)	T_c_/(°C)	*ΔH_m_*/(J/g)	*X_c_*/%
LDPE	108	94	113.5	38.73
LLDPE	121	107	115.6	39.45
MDPE	124	113	141.7	48.36
HDPE	125	113	149.9	51.17

**Table 2:** Shape Parameter and Breakdown Strength of Polyethylene.

Material	Shape Parameter	Characteristic Breakdown Strength (kV/mm)
LDPE	7.791	328
LLDPE	5.277	377
MDPE	6.654	422
HDPE	9.577	453

**Table 3:** The slope of the line in different areas of different materials.

Different Areas	T1	T2	T3
LDPE	1.16	3.94	2.13
LLDPE	1.42	2.93	1.23
MDPE	1.36	2.89	0.89
HDPE	1.15	2.83	1.12

**Table 4:** The corresponding field strength at different turning points of different materials.

The Turning Point	LDPE	LLDPE	MDPE	HDPE
A	12.76 kV/mm	13.33 kV/mm	15.68 kV/mm	18.37 kV/mm
B	50.88 kV/mm	53.61 kV/mm	65.34 kV/mm	71.89 kV/mm
